# Predomination and New Genotypes of *Enterocytozoon bieneusi* in Captive Nonhuman Primates in Zoos in China: High Genetic Diversity and Zoonotic Significance

**DOI:** 10.1371/journal.pone.0117991

**Published:** 2015-02-23

**Authors:** Md Robiul Karim, Haiju Dong, Tongyi Li, Fuchang Yu, Dezhong Li, Longxian Zhang, Junqiang Li, Rongjun Wang, Shouyi Li, Xiaofeng Li, Farzana Islam Rume, Changshen Ning

**Affiliations:** 1 College of Animal Science and Veterinary Medicine, Henan Agricultural University, Zhengzhou, 450002, China; 2 Zhengzhou zoo, Zhengzhou, 45000, China; 3 Department of Microbiology, Patuakhali Science and Technology University, Patuakhali, 8602, Bangladesh; University of Brighton, UNITED KINGDOM

## Abstract

To appreciate the genetic diversity and zoonotic implications of *Enterocytozoon bieneusi* in nonhuman primates (NHPs) in zoos, we genotyped *E. bieneusi* in captive NHPs in seven zoos located at six major cities in China, using ribosomal internal transcribed spacer (ITS)-based PCR and sequence analyses. A total of 496 fecal specimens from 36 NHP species under nine families were analyzed and *E. bieneusi* was detected in 148 (29.8%) specimens of 25 NHP species from six families, including Cercopithecidae (28.7%), Cebidae (38.0%), Aotidae (75.0%), Lemuridae (26.0%), Hylobatidae (50.0%) and Hominidae (16.2%) (P = 0.0605). The infection rates were 29.0%, 15.2%, 18.2%, 37.3%, 29.2%, 37.7% and 44.8% in Shijiazhuang Zoo, Wuhan Zoo, Taiyuan Zoo, Changsha Wild Animal Zoo, Beijing Zoo, Shanghai Zoo and Shanghai Wild Animal Park, respectively (*P* = 0.0146). A total of 25 ITS genotypes were found: 14 known (D, O, EbpC, EbpA, Type IV, Henan-IV, BEB6, BEB4, Peru8, PigEBITS5, EbpD, CM1, CM4 and CS-1) and 11 new (CM8 to CM18). Genotype D was the most prevalent one (40/148), followed by CM4 (20/148), CM1 (15/148), O (13/148), CM16 (13/148), EbpC (11/148). Of them, genotypes D, EbpC, CM4 and O were widely distributed in NHPs (seen in 9 to 12 species) whereas genotypes CM1 and CM16 were restricted to one to three NHP species. In phylogenetic analysis, 20 genotypes (121/148, 81.8%), excluding genotypes BEB4, BEB6, CM9, CM4 and CM18, belonged to group 1 with zoonotic potential. New genotype CM9 clustered in group 2 with BEB4 and BEB6. The remaining two genotypes CM4 and CM18 formed new cluster (group 9) in between two other genotypic clusters found in primates. The findings of high diversity in *E. bieneusi* genotypes and their zoonotic potentiality concluded the importance of captive NHPs as reservoir hosts for human microsporidiosis.

## Introduction

*Enterocytozoon bieneusi*, the dominant member of the human pathogenic microsporidian species, is a unicellular organism that infects the enterocytes of the small intestine and causes diarrhea and enteric diseases in humans, and domestic and wild animals [1–3]. In humans, it has been reported to cause self limiting infections in immunocompetent individuals while life-threatening chronic diarrhea and wasting diathesis in immunocompromised patients, particularly in AIDS patients and organ transplant recipients [1,4–6].

Since its first identification in AIDS patient by Desportes and others in 1985, molecular studies in various hosts and water bodies revealed *E*. *bieneusi* as potentially zoonotic pathogen worldwide [1–3,6–17]. However, the reservoir hosts of this pathogen and their precise role in zoonotic transmission are poorly understood [1,3,17–20]. Internal transcribed spacer (ITS) based genotyping and phylogenetic analysis help us to evaluate host specificity and the zoonotic potential of *E*. *bieneusi* [1,21]. With constant identification of new ITS genotypes, thus far, over 200 *E*. *bieneusi* genotypes have been reported in various animals, humans and water bodies worldwide [1–3,16,17,22–25]. In phylogenetic analysis, the genotypes constitute at least nine distinct genetic clusters (groups 1 to 8 and the so-called outlier in dog) [3]. Due to likely transmission between humans and animals, the large number of genetically related genotypes with broad host ranges form group 1 of zoonotic potentials. Whereas, groups 2 to 5, 8 and the outlier chiefly consist of genotypes those are animal host-adapted. The remaining groups 7 and 6 containing the genotypes those are found in AIDS patients in Nigeria, and urban wastewater in China, respectively [3,9,16,21].

Data from recent molecular studies in China and Kenya regarding *E*. *bieneusi* in nonhuman primates (NHPs) report that the pathogen is commonly found in different NHPs with its considerable genetic diversity. In those studies, a total of 29 *E*. *bieneusi* ITS genotypes have been reported in various species of NHPs [3,11,15,20,26]. The majority of the genotypes were found to be potentially zoonotic ones belonging to genotypic group 1 and some of these such as genotypes A, D, Type IV, EbpC, Peru7, Peru8, Peru11, PigEBITS7, Henan-V, WL15, I and BEB6 (reported as SH5 in children by Wang and others in 2013 [14]), have been detected to infect humans in many parts of the world including China [1,2,8,12–14]. These observations raise deep concern on the importance of NHPs in the zoonotic and/or anthroponotic transmission of *E*. *bieneusi* worldwide. Despite the emerging potential importance of NHPs in human microsporidiosis by *E*. *bieneusi*, studies in these closest relatives of us yet remain scarce.

In zoo, there is either possibility of *E*. *bieneusi* transmission from infected NHPs to animal caretakers and visitors or from infected people to NHPs through water and/or food contamination. Therefore, it is notably important to better understand the molecular epidemiological relevance to the transmission patterns of this parasitic pathogen in and from zoo facilities. Considering the possibility and importance, we have examined *E*. *bieneusi* in 163 NHP fecal specimens from three zoos along with 1,223 specimens from monkey farms, research laboratory and free range NHPs and observed comparatively lower occurrence in zoo specimens with only three known genotypes in four NHP species surprisingly in our previous study [3]. These results raised a question whether the natural infection and circulating genotypes of *E*. *bieneusi* are really low in NHPs in zoos or not and drew our attention to further investigate this parasite in other zoo NHPs to resolve the question. Thus, the present study was designed to investigate the occurrence of natural infection of *E*. *bieneusi* in NHPs kept in seven different zoos in China using molecular analysis, to determine the diversity in circulating *E*. *bieneusi* genotypes and their zoonotic potential by comparing the ITS gene sequences obtained with those from GenBank.

## Materials and Methods

### Ethics statement

This research was carried out likewise the Chinese Laboratory Animal Administration Act 1988 after reviewing and approving its protocol by the Research Ethics Committee of Henan Agricultural University. Appropriate permission was obtained from zoo owners before collection of fecal specimens from NHPs.

### Study sites and sampling

**Fig 1:**
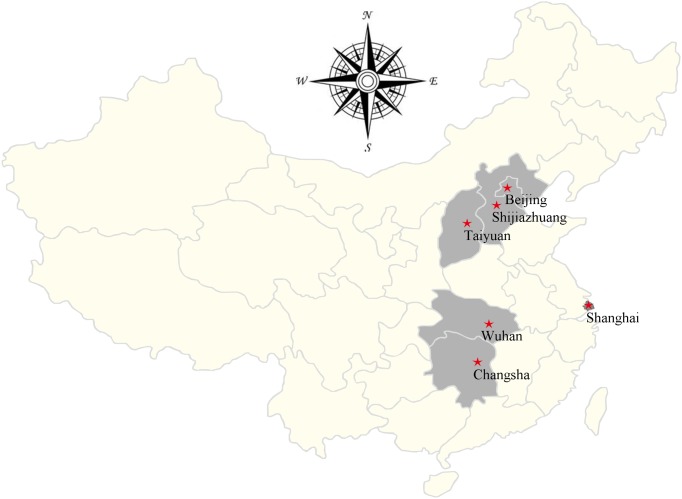
Specific locations at which specimens were collected in this study. ★locations of zoos.

The study was conducted for a four-month period during the winter season (October 2013 to January 2014) in Beijing Zoo (located at Northern China), Shanghai Zoo, Shanghai Wild Animal Park (at East China), Changsha Wild Animal Zoo (at the capital of South-central China’s Hunan Province), Wuhan Zoo (at the capital of Central China’s Hubei Province), Shijiazhuang Zoo (at the capital of North China’s Hebei Province) and Taiyuan Zoo (at the capital of North China’s Shanxi Province) (Fig. 1 and Table 1). All the zoos are accredited and owned by the government of PR China. In the zoos, the animals are housed in large spaces with reconstruction of natural habitats suitable for each species. The NHPs in general live in accommodations provided with shelter places and artificial hills. The NHPs are kept separately according to the species in all the zoos. Some of the animals live individually in single cages while others live in groups. To avoid unusual contact with visitors, glass screens and ditches are used as boundary of the cages and pens. The NHPs are commonly fed bread, vegetables, seasonal fruits and peanuts offered twice a day, in the morning and afternoon.

**Table 1:** Prevalence and ITS genotypes distribution of *E*. *bieneusi* in nonhuman primates in different zoos in China.

Study location	No. of specimens	No. (%) of positive specimens	ITS genotypes (no. of specimens)		
			Zoonotic	Potentially zoonotic	Others
Shijiazhuang Zoo, Hebei Province	89	24 (27.0)	Type IV (3), Henan-IV (2), D (1), EbpC (1), EbpA (1)	CM1 (15), CM8 (1)	
Wuhan Zoo, Hubei Province	66	10 (15.2)	D (5), EbpC (3), BEB6 (2)		
Taiyuan Zoo, Shanxi Province	66	12 (18.2)	D (6), Henan-IV (1)		CM4 (4), CM9 (1)
Changsha Wild Animal Zoo, Hunan Province	75	28 (37.3)	D (15), EbpC (4), O (3), Type IV (1), BEB6 (1)	CM12 (2), CM13 (1), CM14 (1)	
Beijing Zoo	72	21 (29.2)	O (8), EbpA (4), EbpC (2), Type IV (1), EbpD (1), Peru8 (1), PigEBITS5 (1),	CM10 (1), CM11 (1), CS-1 (1)	
Shanghai Zoo	61	23 (37.7)	D (4), O (2), EbpA (1), Henan-IV (1)	CM15 (1), CM16 (1)	CM4 (13),
Shanghai Wild Animal Park	67	30 (44.8)	D (9), BEB4 (2), EbpC (1)	CM16 (12), CM17 (2),	CM4 (3), CM18 (1)

A total of 496 fresh fecal specimens were collected from 36 NHP species under nine families, including Cercopithecidae, Pitheciidae, Cebidae, Atelidae, Aotidae, Lorisidae, Lemuridae, Hylobatidae and Hominidae, kept in the zoos (Tables 1 and 2). In case of group housing, fresh fecal deposits were collected in the early morning, since the floor of animal cages was cleaned every evening. The fresh fecal deposits were selected according to their color and consistency. For animals that were kept in the pens during the day, fecal specimens were collected from individual boxes where they spent the night. The specimens were collected with the help of respective animal attendants to minimize unnecessary fear due to strangers (collectors) in the houses. The fecal specimens were placed into clean plastic bags marked with relative information, shipped in cool condition to the Laboratory of Veterinary Parasitology, Henan Agricultural University, transferred in water into a 50-ml centrifuge tube, sieved through a 7.62-cm-diameter sieve with a pore size of 45 μm and concentrated by centrifugation. The concentrated fecal specimens were then stored in 2.5% potassium dichromate solution at 4°C until DNA extraction.

**Table 2:** Infection rate and genotypes of *E*. *bieneusi* in nonhuman primates based on PCR and sequence analysis of ITS locus.

NHP Family/Scientific name (Common name)	No. of specimens tested	No. (%) of positive specimens	ITS genotypes (no. of specimens)		
			Zoonotic	Potentially zoonotic^a^	Others
Cercopithecidae	289	83 (28.7)			
*Macaca mulatta* (rhesus macaque)	106	33 (31.1)	D (13), BEB6 (2), O (1), EbpC (1)	CM1 (15), CM8 (1)	
*Macaca assamensis* (Assam macaque)	3	0			
*Macaca fascicularis* (cynomolgus monkey)	18	5 (27.8)	EbpC (1), BEB6 (1)	CM12 (2), CM13 (1)	
*Macaca nemestrina* (pig-tailed macaque)	16	4 (25.0)	O (1), EbpC (1), PigEBITS5 (1)	CM14 (1)	
*Macaca thibetana* (Tibetan macaque)	2	0			
*Macaca nigra* (celebes crested macaque)	2	1 (50.0)	O (1)		
*Erythrocebus patas* (hussar monkey)	16	8 (50.0)	D (5), O (2), EbpC (1)		
*Cercopithecus roloway* (roloway monkey)	1	0			
*Trachypithecus francoisi* (Francois’ leaf monkey)	10	3 (30.0)	EbpA (1)	CM15 (1)	CM4 (1)
*Cercopithecus kandti* (golden monkey)	29	9 (31.0)	D (8)		CM4 (1)
*Colobus polykomos* (king colobus)	7	1 (14.3)	D (1)		
*Cercopithecus mona* (mona monkey)	6	0			
*Cercopithecus neglectus* (De Brazza’s monkey)	3	0			
*Cercopithecus diana* (Diana monkey)	1	0			
*Chlorocebus sabaeus* (green monkey)	15	10 (66.7)	D (7), Type IV (1)	CS-1 (1)	CM4 (1)
*Mandrillus sphinx* (mandrill)	23	1 (4.4)			CM4 (1)
*Papio hamadryas* (hamadryas baboon)	21	6 (28.6)	D (1), Peru8 (1), EbpC (1), Henan-IV (1)		CM4 (2)
*Papio cynocephalus* (yellow baboon)	5	0			
*Papio anubis* (olive baboon)	5	2 (40.0)	D (1)		CM4 (1)
Pitheciidae	2	0			
*Chiropotes satanas* (black bearded saki)	2	0			
Cebidae	79	30 (38.0)			
*Cebus apella* (black-capped capuchin)	22	6 (27.3)	O (2), EbpA (1), EbpD (1), BEB4 (1)	CM16 (1)	
*Cebus olivaceus* (weeper capuchin	4	3 (75.0)	O (1), EbpC (1), EbpA (1)		
*Cebus albifrons* (white-fronted capuchin)	5	1 (20.0)	O (1)		
*Saimiri* sp. (squirrel monkey)	43	17 (39.5)	O (1), EbpC (1), EbpA (1)	CM16 (10)	CM4 (4)
*Callithrix* sp. (marmoset)	5	3 (60.0)			CM4 (3)
Atelidae	5	0			
*Ateles paniscus* (black spider monkey)	5	0			
Aotidae	4	3 (75.0)			
*Aotus* sp. (night monkey/owl monkey)	4	3 (75.0)			CM4 (3)
Lorisidae	4	0			
*Nycticebus coucang* (sunda slow loris)	4	0			
Lemuridae	50	13 (26.0)			
*Lemur catta* (ring-tailed lemur)	45	11 (24.4)	Type IV (4), EbpA (1), O (1)	CM16 (2), CM10 (1), CM11 (1)	CM18 (1)
*Varecia variegata* (black-and-white ruffed lemur)	5	2 (40.0)	EbpC (1), O (1)		
Hylobatidae	26	13 (50.0)			
*Nomascus leucogenys* (northern white-cheeked gibbon)	14	5 (35.7)	Henan-IV (3), D (1), O (1)		
*Hylobates moloch* (silvery gibbon)	3	3 (100)	EbpC (2)		CM4 (1)
*Hylobates lar* (white-handed gibbon)	8	5 (62.5)	EbpC (1), EbpA (1), BEB4 (1)	CM17 (2)	
*Hylobates pileatus* (pileated gibbon)	1	0			
Hominidae	37	6 (16.2)			
*Pongo pygmaeus* (bornean orangutan)	23	4 (17.4)	D (3)		CM4 (1)
*Pan troglodytes* (common chimpanzee)	14	2 (14.3)			CM4 (1), CM9 (1)
Total	496	148 (29.8)	D (40), O (13), EbpC (11), EbpA (6), Type IV (5), Henan-IV (4), BEB6 (3), BEB4 (2), Peru8 (1), PigEBITS5 (1), EbpD (1)	CM1 (15), CM16 (13), CM12 (2), CM17 (2), CM8 (1), CM10 (1), CM11 (1), CM13 (1), CM14 (1), CM15 (1), CS-1 (1)	CM4 (20), CM9 (1), CM18 (1)

^a;^ Genotypes belonged to group 1 in phylogenetic analysis with having potentiality to infect both humans and animals

### DNA extraction

The preserved fecal specimens were washed three times with distilled water by centrifugation to remove the potassium dichromate. Genomic DNA was extracted using the E.Z.N.A.R Stool DNA kit (Omega Biotek Inc., Norcross, USA) according to manufacturer-recommended protocols. The extracted DNA was stored at −20°C until used in PCR analysis.

### PCR detection and nucleotide sequencing

*E*. *bieneusi* was detected by nested PCR amplification of a 390-bp product of the partial 18S rRNA gene, the complete internal transcribed spacer (ITS), and the partial 5.8S rRNA gene. The primers used included EBITS3 (5′-GGTCATAGGGATGAAGAG-3′) and EBITS4 (5′-TTCGAGTTCTTTCGCGCTC-3′) for primary PCR and EBITS1 (5′-GCTCTGAATATCTATGGCT-3′) and EBITS2.4 (5′-ATCGCCGACGGATCCAAGTG-3′) for secondary PCR. The reaction conditions were described in previous study [27]. Each specimen was analyzed twice by using 2 μl of extracted DNA per PCR performed in an Applied Biosystems 2720 Thermal Cycler (Applied Biosystems, Foster City, USA). The rTaq amplification enzyme (Takara Biotechnology Co. Ltd. Dalian, China) was used for PCR amplification. Non-acetylated bovine serum albumin (Solarbio Co. Ltd, Beijing, China) at a concentration of 400 ng/μl was used in the primary PCR to neutralize the PCR inhibitors. The secondary PCR products were examined by agarose gel electrophoresis and visualized after GelRed (Biotium Inc., Hayward, CA) staining.

After purification using Montage PCR filters (Millipore, Bedford, MA), positive secondary PCR products were directly sequenced with the secondary PCR primers using the Big Dye Terminator v3.1 Cycle Sequencing kit (Applied Biosystems) on an ABI 3730 DNA Analyzer (Applied Biosystems, Foster City, USA). The nucleotide sequence accuracy was confirmed by two-directional sequencing and by sequencing a new PCR product if necessary.

### Sequence and genetic proximity analysis

The obtained sequences were aligned with reference sequences downloaded from GenBank using the program ClustalX 1.83 (http://www.clustal.org/) to determine genotypes. The genetic proximity of the genotypes from this study was compared with previously reported *E*. *bieneusi* ITS genotypes using a neighbor-joining analysis based on genetic distances calculated by the Kimura two-parameter model implemented in the program Mega 5 (http://www.megasoftware.net/). Bootstrap analysis was used to assess the robustness of clusters using 1,000 replicates. The established nomenclature system was used in the naming of *E*. *bieneusi* ITS genotypes [28].

### Statistical analysis

The differences in infection rates among NHP families and zoos were compared using the chi-square test implemented in the software QuickCalcs (GraphPad Software Inc., La Jolla, CA). A difference was considered significant when the *P* value was <0.05.

### Nucleotide sequence accession numbers

Representative nucleotide sequences from this study were deposited in the GenBank under accession numbers KJ728787 to KJ728811.

## Results

### Occurrence of *E*. *bieneusi*

Based on ITS-PCR amplification, 148 (29.8%) specimens were positive for *E*. *bieneusi* out of the 496 NHP specimens examined. The pathogen was detected in six NHP families, including Cercopithecidae (28.7%, 83/289), Cebidae (38.0%, 30/79), Aotidae (75.0%, 3/4), Lemuridae (26.0%, 13/50), Hylobatidae (50.0%, 13/26) and Hominidae (16.2%, 6/37), of the nine families studied. The differences in infection rates among NHP families were not statistically significant (*P* = 0.0605). Twenty five of 36 NHP species under the families were found to be infected with *E*. *bieneusi* with the occurrences ranging from 4.4 to 100%. (Table 2). Twenty four of 89 (29.0%) specimens from Shijiazhuang Zoo, ten of 66 (15.2%) specimens from Wuhan Zoo, twelve of 66 (18.2%) specimens from Taiyuan Zoo, twenty eight of 75 (37.3%) specimens from Changsha Wild Animal Zoo, twenty one of 72 (29.2%) specimens from Beijing Zoo, twenty three of 61 (37.7%) specimens from Shanghai Zoo and thirty of 67 (44.8%) specimens from Shanghai Wild Animal Park were identified to be *E*. *bieneusi* positive (Table 1). The differences in occurrences among seven zoos were statistically significant (*P* = 0.0146).

### *E*. *bieneusi* ITS genotypes

In the ITS nucleotide sequence analysis, a total of 25 distinct *E*. *bieneusi* genotypes, including 14 previously reported or known genotypes (D, O, EbpC, EbpA, Type IV, Henan-IV, BEB6, BEB4, Peru8, PigEBITS5, EbpD, CM1, CM4 and CS-1) and 11 new genotypes (named CM8 to CM18 in this study), were determined among the 148 positive specimens. Genotype D was the most common one (40/148, 27.0%), followed by CM4 (20/148, 13.5%), CM1 (15/148, 10.1%), O (13/148, 8.8%), CM16 (13/148, 8.8%), EbpC (11/148, 7.4%), EbpA (6/148, 4.1%), Type IV (5/148, 3.4%), Henan-IV (4/148, 2.7%) and BEB6 (3/148, 2.0%). Genotypes BEB4, CM12 and CM17 were found in two specimens (2/148, 1.4%) each while the remaining 12 genotypes were seen in one specimen (1/148, 0.7%) each. Among the prevalent genotypes, CM4, O, EbpC and D were more widely distributed in the species of NHPs, being detected in 12, 11, 10 and 9 species, respectively whereas genotypes CM1 and CM16 were restricted to one and three NHP species, respectively (Table 2).

The highest sequence polymorphism was found in the Cercopithecidae family, with 17 ITS genotypes being observed in 83 positive specimens. The major genotypes were D (*n* = 36) and CM1 (*n* = 15). Fifteen other genotypes found in this family were CM4 (*n* = 7), O (*n* = 5), EbpC (*n* = 5), BEB6 (*n* = 3), CM12 (*n* = 2), PigEBITS5 (*n* = 1), EbpA (*n* = 1), Type IV (*n* = 1), Peru8 (*n* = 1), Henan-IV (*n* = 1), CS-1 (*n* = 1), CM8 (*n* = 1), CM13 (*n* = 1), CM14 (*n* = 1) and CM15 (*n* = 1). The NHPs under Cebidae family were infected with genotypes CM16 (*n* = 11), CM4 (*n* = 7), O (*n* = 5), EbpA (*n* = 3), EbpC (*n* = 2), EbpD (*n* = 1) and BEB4 (*n* = 1). Only one genotype, CM4 (*n* = 3) was identified in Aotidae family. In the animals of Lemuridae family, eight genotypes such as Type IV (*n* = 4), O (*n* = 2), CM16 (*n* = 2), EbpC (*n* = 1), EbpA (*n* = 1), CM10 (*n* = 1), CM11 (*n* = 1) and CM18 (*n* = 1) were reported. The animals of Hylobatidae family were infected with genotypes EbpC (*n* = 3), Henan-IV (*n* = 3), CM17 (*n* = 2), D (*n* = 1), O (*n* = 1), EbpA (*n* = 1), BEB4 (*n* = 1) and CM4 (*n* = 1). Three genotypes, including D (*n* = 3), CM4 (*n* = 2) and CM9 (*n* = 1), were found to infect Hominidae family.

The distributions of *E*. *bieneusi* ITS genotypes in seven studied zoos are shown in Table 1. The dominant genotype in Shijiazhuang Zoo was CM1, being detected in 15 specimens, whereas the other six genotypes were detected in 1 to 3 specimens. In Wuhan Zoo, three genotypes D, EbpC and BEB6 were found in 5, 3 and 2 specimens, respectively. Genotypes D (*n* = 6) and CM4 (*n* = 4) were observed as major ones in Taiyuan Zoo; other two genotypes each found in single specimen. The NHPs in Changsha Wild Animal Zoo were mostly infected with genotype D (*n* = 15). The remaining seven genotypes identified in this zoo were seen in 1 to 4 specimens. The largest number (10) of genotypes was noticed in Beijing Zoo and the prevalent genotypes were O (*n* = 8) and EbpA (*n* = 4). The other eight genotypes were seen in 1 to 2 specimens. In Shanghai Zoo, the common genotype CM4 was found in 13 specimens, whereas genotypes D, O, EbpA, Henan-IV, CM15 and CM16 were seen in 1 to 4 specimens. In contrast, genotypes CM16 (*n* = 12) and D (*n* = 9) were predominant in NHPs in Shanghai Wild Animal Park. Other genotypes included CM4 (*n* = 3), BEB4 (*n* = 2), CM17 (*n* = 2), EbpC (*n* = 1) and CM18 (*n* = 1) (Table 1).

### Phylogenetic analysis and genetic proximity

**Fig 2:**
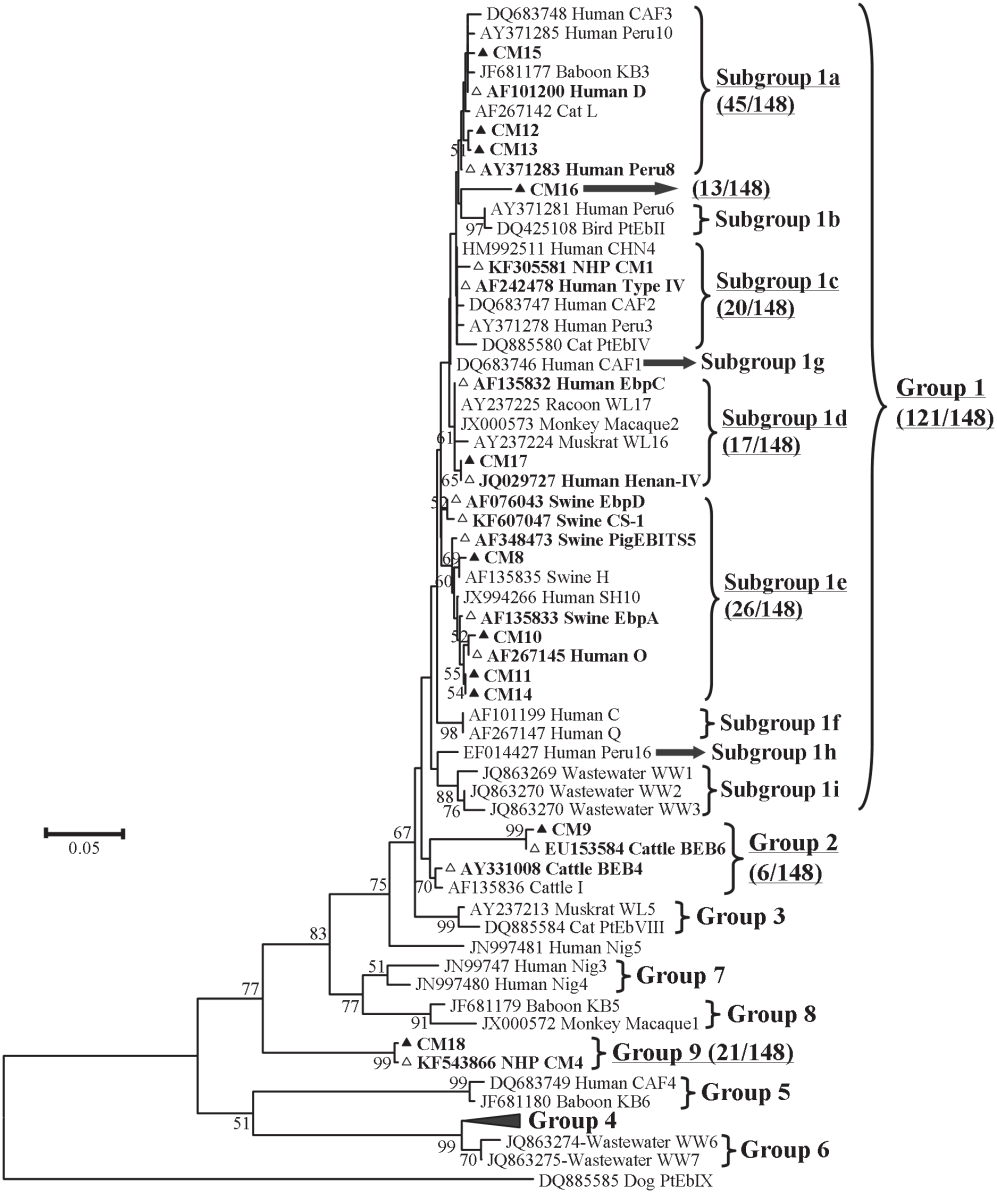
Neighbor-joining tree of *E*. *bieneusi* ITS genotypes. Phylogenetic relationship of *E*. *bieneusi* ITS nucleotide sequences of this study and other genotypes previously deposited in GenBank, as inferred by a neighbor-joining analysis (software Mega 5, http://www.megasoftware.net/) based on genetic distances calculated using the Kimura two-parameter model. The ITS tree was rooted with GenBank sequence DQ885585. Bootstrap values greater than 50% from 1,000 are shown on nodes. Each sequence from GenBank is identified by the accession number, host origin, and the genotype designation. The group terminology for the clusters is based on the works of Thellier and Breton [21], Li et al. [9], and Karim et al. [3]. Two unique sequences of new genotype CM18 and known genotype CM4 in this study are designated as group 9 sequences. Known and new genotypes identified in this study are indicated by open and filled triangles, respectively.

Of the 25 ITS genotypes found in this, 20 (121/148, 81.8%) belonged to previously described zoonotic group 1 in phylogenetic analysis (Fig. 2). Three genotypes such as BEB6, BEB4 and CM9 seen in six animals (6/148, 4.1%) were clustered in so-called bovine specific group 2 [21,29,30]. The remaining two genotypes (CM4 and CM18) identified in 21 animals (21/148, 14.2%) formed new cluster (named as group 9 in this study) in between groups 5 and 8 [3] (Fig. 2). The distributions of observed known zoonotic genotypes, potentially zoonotic genotypes (genotypes of group 1 with potentiality to infect humans) and others in NHP species and in zoos in this study are presented in Tables 1 and 2.

Nucleotide sequence analysis revealed that the new genotypes CM12 and CM15 had one single nucleotide polymorphism (SNP) at nucleotide positions 105 (G-to-T) and 158 (A-to-G), respectively compared to genotype D (KF305583); and genotype CM13 had one SNP at position 105 (G-to-T) compared to Peru8 (KF305584). These three genotypes clustered in subgroup 1a. Genotype CM17 had one SNP at position 88 (G-to-A) compared to genotype EbpC (AB470284) and thus located in subgroup 1d. Genotypes CM11 and CM14 had two SNPs at positions 179 (T-to-G) and 202 (G-to-A); and at 202 (G-to-A) and 352 (G-to-A), respectively compared to genotype SH10 (JX994266). Genotype CM10 had one SNP at position 274 (G-to-A) compared to genotype O (AF267145), and genotype CM8 had two SNPs at positions 171 (G-to-A) and 303 (G insertion) compared to genotype H (AF135835). Hence, these four genotypes (CM8, CM10, CM11 and CM14) grouped in subgroup 1e. The remaining new genotype CM16 in group 1 being detected in 13 animals did not cluster in any of the previous subgroups but was sister to subgroup 1b (Fig. 2). It had eight SNPs compared to genotype Peru8 (KF305584). On the other hand, genotype CM9 in group 2 had one SNP at position 325 (G-to-A) compared to BEB6 (KF543869). The genotype CM18 had two SNPs at positions 76 (A-to-G) and 281 (T-to-C) compared to genotype CM4 (KF543866) and therefore, they together formed new genotypic group 9.

## Discussion

In this study, *E*. *bieneusi* was detected in 29.8% (148/496) of the NHP fecal specimens analyzed, which illustrates its common occurrence in NHPs. The infection was noticed in NHPs of 25 species under six families housed in all the seven studied zoos in China. A similar infection rate (28.2%) of *E*. *bieneusi* was recorded in rhesus macaques in a public park in Guiyang city in China [11]. However, little lower infection rates of 18.2% and 12.3% were reported in laboratory cynomolgus monkeys in Guangxi, China [15] and captive baboons in Kenya [26], respectively. Furthermore, the overall infection rate of *E*. *bieneusi* was 11.4% in different NHP species from various parts of China examined in our previous study [3]. The study also reported the infection rates of 13.7% and 5.0% in captive and free range NHPs, respectively. In captive NHPs, the infection rates were recorded as 26.5%, 13.0% and 7.4% in research laboratory, monkey farms and zoos, respectively [3]. The findings of the present study along with the previous observations illustrate the fact that *E*. *bieneusi* is a prevalent pathogen in NHPs particularly in China. The results on infections of *E*. *bieneusi* in 25 NHP species of six families here with the rates ranging from 4.4 to 100% are comparable with previous studies. In our recent report, only five NHP species (all from Cercopithecidae family) among 23 species were positive for *E*. *bieneusi* [3]. In rhesus macaques, the 31.1% infection rate reported in this study is higher than the previous reports [3,11], whereas in cynomolgus monkeys, the prevalence (27.8%) in this report is within the range (18.5% to 67.7%) of prevalence recorded in previous studies [3,15]. The infection rate of 25.8% in baboons here is higher than the infection rate (12.3%) reported by Li and associates [26] in captive baboons in Kenya. This relatively higher prevalence of *E*. *bieneusi* infection covering wide NHP host species in this study indicates that NHPs kept in zoos are commonly infected with the parasitic pathogen, which might resolve the question regarding the actual occurrence of *E*. *bieneusi* infection in zoo NHPs in our previous study. The lower prevalence in zoos in our previous study could be either due to smaller number of specimens examined, low PCR amplification efficiency of the primers used in the specimens or low parasite burden of the animals sampled.

The present research determined a high degree of genetic diversity in *E*. *bieneusi* from zoo NHPs. A total of 25 explicit ITS genotypes (14 known and 11 new) were observed in 148 positive animals. As compared to our previous findings on genotypes in zoo NHPs [3], the wide range of genetic polymorphisms in this study elaborates our understanding about circulating genotypes in NHPs in zoos. Among the known genotypes in this study, 11 including genotypes D, O, EbpC, EbpA, Type IV, Henan-IV, BEB4, Peru8, PigEBITS5, EbpD and BEB6 (reported as SH5) have been detected in humans in many countries in the world [1,2,8,12–14]. Despite the lack of data regarding transmission of the parasite whether it is anthroponotic or zoonotic, the findings of higher number of human infective genotypes in this study further illustrate the potential role of NHPs in human microsporidiosis by *E*. *bieneusi* worldwide [3]. Besides, all the 14 known genotypes have been reported in HIV-positive and-negative persons, children, NHPs, pigs, cattle, sheep, dogs, cats, snakes and urban wastewater in different locations in China (Table 3). This observation suggests that captive NHPs could be the key reservoir hosts of prevailing *E*. *bieneusi* genotypes in China, majority of which were reported to cause human microsporidiosis and water contamination in some areas of this country [9,13,14].

**Table 3:** Summary on the distribution of known *E*. *bieneusi* genotypes of this study in China.

Genotype	Host/source (*location* ^b^)	References
D	HIV-positive and-negative person (*Henan*), Children (*Shanghai*), NHP (*Henan*, *Guangxi*, *Guangdong*, *Yunnan*, *Sichuan*), Pig (*Heilongjiang*), Wastewater (*Shanghai*, *Nanjing*, *Qingdao*, *Wuhan*), Dog (*Henan*), Cat (*Henan*)	[3,9,13,14,15,17,23,24]
O	Pig (*Heilongjiang*, *Jilin*, *Inner Mongolia*), Dog (*Henan*)	[17,23,24]
EbpC	HIV-positive and-negative human (*Henan*), Children (*Shanghai*), NHP (*Henan*, *Guizhou*), Pig (*Heilongjiang*, *Jilin*, *Inner Mongolia*), Wastewater (*Shanghai*, *Qingdao*, *Wuhan*), Dog (*Shaanxi*)	[3,9,11,13,14,17,22,24]
EbpA	Children (*Shanghai*), Pig (*Heilongjiang*, *Jilin*), Wastewater (*Wuhan*), Dog (*Henan*)	[14,17,22,23,24]
Type IV	HIV-positive and-negative human (*Henan*), NHP (*Henan*, *Guangxi*, *Guangdong*, *Yunnan*, *Sichuan*, *Guizhou*), Wastewater (*Shanghai*, *Qingdao*, *Wuhan*), Dog (*Henan*), Snake (*Guangxi*)	[3,9,11,13,24,25]
EbpD	HIV-positive human (*Henan*), Pig (*Heilongjiang*, *Jilin*), Wastewater (*Qingdao*)	[9,13,17,22]
Henan-IV	HIV-positive human (*Henan*), Pig (*Heilongjiang*)	[13,17]
BEB6^a^	Children (*Shanghai*), NHP (*Sichuan*), Wastewater (*Qingdao*, *Wuhan*), Sheep (*Heilongjiang*), Cat (*Henan*)	[3,9,14,22,24]
BEB4^a^	Human, Cattle, Pig (*Jilin*)	[32]
Peru8	HIV-positive human (*Henan*), NHP (*Guangxi*, *Guangdong*, *Yunnan*), Wastewater (*Shanghai*, *Nanjing*, *Wuhan*), Dog (*Henan*)	[3,9,13,24]
CS-1	Pig (*Heilongjiang*, *Jilin*)	[17,23]
CM1	NHP (*Guangdong*, *Guangxi*, *Yunnan*, *Sichuan*), Dog (*Henan*)	[3,24]
CM4	NHP (*Henan*, *Sichuan*)	[3]
PigEBITS5	Dog (*Henan*)	[24]

^a;^ Genotypes BEB6 and BEB4 reported as SH5 and CHN1 in the studies by Wang et al. [14] and Zhang et al. [32]. NHP: Nonhuman primate

^b;^ Province or city in China

The zoonotic genotype D, the most common one in this study, has also been identified as prevalent genotype in humans and many animal species worldwide [1,2,6,12]. In China, it was found to be dominant genotype in NHPs, pigs, cats and urban wastewater [3,9,23,24]. Additionally, the other prevalent genotype EbpC in this study was previously identified as frequent cause of microsporidiosis in HIV-positive and-negative persons, and in pigs in China [13,17]. However, the genotype CM1, one of the major genotypes here, was only reported in NHPs and found to be dominant in our preceding study [3]. In the light of this observation, it is worth noting here that the two prevalent *E*. *bieneusi* genotypes D and EbpC could pose public health concerns in China where NHPs could play significant role.

In phylogenetic analysis, nine of the 11 new *E*. *bieneusi* genotypes (CM8 and CM10 to CM17) in this study are genetically related to the genotypes found in group 1 [21] and thus have zoonotic potential and public health importance. Of them, genotypes CM12 and CM15 are closely related to genotype D with one SNP, and CM13 resembles to genotype Peru8 with one SNP. Thus, they are placed in subgroup 1a. Genotypes CM11 and CM14 have two nucleotide substitutions compared to genotype SH10 identified in children in China [14]. Conversely, genotype CM10 has one SNP compared to genotype O, and genotype CM8 has two SNPs compared to genotype H. Hence, these four genotypes (CM8, CM10, CM11 and CM14) cluster in subgroup 1e. Genotype CM17 in subgroup 1d has one nucleotide difference from genotype EbpC. The remaining new member (genotype CM16) of group 1 having eight SNPs compared to genotype Peru8 is not placed in any of the previous subgroups and is sister to subgroup 1b in phylogenetic analysis [21]. The close genetic relationship of the new genotypes CM11, CM12, CM13, CM14, CM15 and CM17 to the human infective genotypes D, Peru8, SH10 and EbpC reported in HIV-positive and-negative persons and children in China, suggests that they are potential human pathogens [13,14]. Of the two new host specific genotypes, CM9 has one SNP comparative to genotype BEB6 and forms a cluster with the genotypes of so-called cattle-specific group 2 [21]. In our recent study, other two genotypes CM5 and CM7 in NHPs belonged to this group 2 [3]. Furthermore, some members of cattle-specific group 2 were found in humans in recent past [2,14]. These observations further suggest that genotypes in group 2 are not cattle specific [3]. The remaining new genotype CM18 has two nucleotide differences from genotype CM4 isolated from NHPs in our previous study [3]. These two genotypes detected in 21 animals form new cluster (named as group 9 in this study) in between primate-specific genotypic group 5 [9], and the group 8 [3] containing genotypes, macaque 1 [11], KB5 (baboon genotype) [26], and horse 2 [31]. These findings imply that the genotypes of three clusters (groups 5, 8 and newly proposed group 9) near to the base of phylogenetic tree have strong preference to NHP hosts.

In conclusion, data of the present study highlight that *E*. *bieneusi* infection is common among NHPs kept in zoos in China. The findings of high diversity in *E*. *bieneusi* ITS genotypes and their zoonotic potentiality suggest that this parasite can be maintained in NHPs which may contribute to zoonotic transmission under some conditions. Additionally, the findings of identical genotypes in NHPs, and in the humans, several other animal species and wastewater in the same geographical areas unravel the possible happening of cross-species transmission of *E*. *bieneusi*. However, the transmission pathways of *E*. *bieneusi*, whether from infected NHPs to animal caretakers and visitors or vise-versa in zoos, have not been assigned in this study, which deserve to be elucidated in further cross-sectional study involving both NHPs in captive sections and humans as well as the environmental samples. On the basis of our present findings, it is worth noting that attempts should be taken by the authorities of captive NHPs to reduce the contact between susceptible human populations and *E*. *bieneusi* infected NHPs and to reduce the water pollution and groundwater contamination by NHPs fecal sources. In addition to those, personal hygiene should be maintained strictly by the animal attendants, animal care specialists, veterinarians and scientists during handing of NHPs in particular, and by the visitors in the NHP residing areas like zoos in general to cut down the zoonotic as well as anthroponotic transmission of *E*. *bieneusi*.
